# Reshaping the binding channel of a novel GH113 family β-mannanase from *Paenibacillus cineris* (PcMan113) for enhanced activity

**DOI:** 10.1186/s40643-022-00505-7

**Published:** 2022-03-05

**Authors:** Dengyue Sun, Chao Li, Pengpeng Cui, Jie Zhang, Yaolin Zhou, Mian Wu, Xia Li, Teng-fei Wang, Zhixiong Zeng, Hui-Min Qin

**Affiliations:** 1 State Key Laboratory of Biobased Material and Green Papermaking, College of Bioengineering, Qilu University of Technology, Shandong Academy of Sciences, Jinan, 250100 People’s Republic of China; 2 College of Biotechnology, Tianjin University of Science and Technology, Tianjin, 300457 People’s Republic of China; 3 School of Bioengineering, Qilu University of Technology, Shandong Province, Jinan, 250353 People’s Republic of China

**Keywords:** Endo**-**β-mannanase, GH113 family, Structural analysis, Binding channel, Molecular dynamics

## Abstract

Endo-β-mannanases are important enzymes for degrading lignocellulosic biomass to generate mannan, which has significant health effects as a prebiotic that promotes the development of gut microbiota. Here, a novel endo-β-mannanase belonging to glycoside hydrolase (GH) family 113 from *Paenibacillus cineris* (PcMan113) was cloned, expressed and characterized, as one of only a few reported GH113 family β-mannanases. Compared to other functionally and structurally characterized GH113 mannanases, recombinant PcMan113 showed a broader substrate spectrum and a better performance. Based on a structural homology model, the highly active mutant PcMT3 (F110E/N246Y) was obtained, with 4.60- and 5.53-fold increases of enzyme activity (towards KG) and catalytic efficiency (*k*_cat_/*K*_m_, against M5) compared with the WT enzyme, respectively. Furthermore, molecular dynamics (MD) simulations were conducted to precisely explore the differences of catalytic activity between WT and PcMT3, which revealed that PcMT3 has a less flexible conformation, as well as an enlarged substrate-binding channel with decreased steric hindrance and increased binding energy in substrate recognition. In conclusion, we obtained a highly active variant of PcMan113 with potential for commercial application in the manufacture of manno-oligosaccharides.

**Graphical Abstract:**

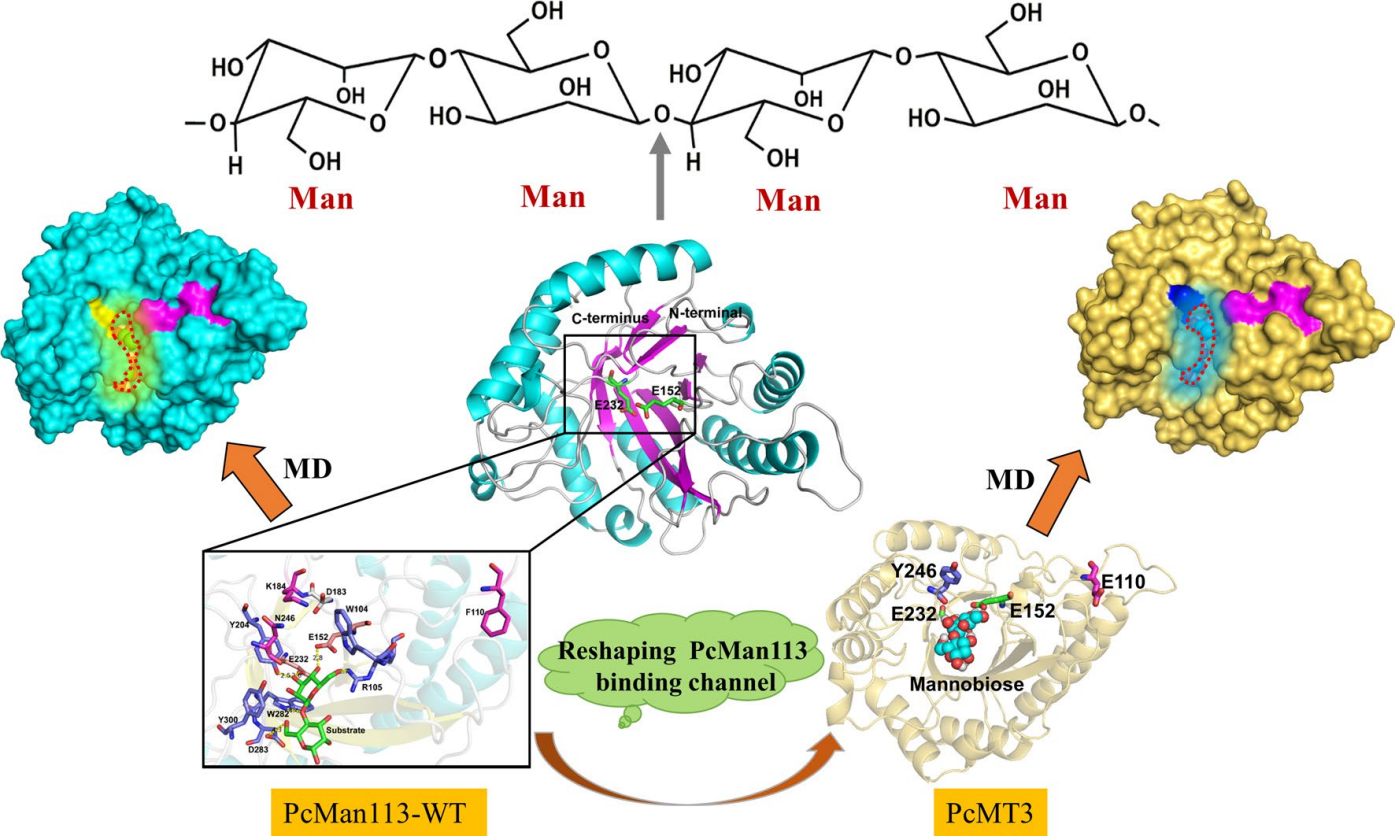

**Supplementary Information:**

The online version contains supplementary material available at 10.1186/s40643-022-00505-7.

## Introduction

Hemicellulose is the second most abundant polysaccharide in nature, and mannan is a major hemicellulose component normally found in the cell walls and seeds of plants (Saha, 2003). The degradation of mannan polysaccharides relies on microbial mannanases, which are primarily endo-type enzymes, and the resulting manno-oligosaccharide (MOS) products are further degraded into monomers, which play important roles in microbial metabolism and plant growth (Srivastava and Kapoor, 2017a, 2005, b; Moreira et al. 2008). Normally, MOS prepared by enzymatic methods are mainly sourced from locust bean gum (LBG), konjac gum (KG), and guar gum (GG) (Malgas et al. 2015).

Mannan is receiving increasing attention due to its health effects mediated by gut microbiota and probiotics (Mano et al. 2018; Pérez-Burillo et al. 2019). MOS act as prebiotics that can also be used in food directly, and have attracted attention from the healthcare industry due to their functional activities, such as promoting the growth of probiotics, inhibiting the growth of pathogenic bacteria and contributing to the health of the intestinal environment (Pérez-Burillo et al. 2019; Zheng et al. 2018; Srivastava et al. 2014; Yamabhai et al. 2016; La Rosa et al. 2019). The practice of using MOS as feed additives can enhance the growth performance of pigs, as well as improve posterior gut epithelial defense, egg production and antibody production efficiency of laying hens (Giannenas et al. 2016; Ghasemian et al. 2016). In addition, MOS have been reported to enhance the immunity and resistance of pacific white shrimp (Torrecillas et al. 2014). Therefore, the demand for MOS is increasing due to their great potential for applications in the feed, food, and pharmaceutical industries (Srivastava et al. 2017a, b; Cao et al. 2018; Rai et al. 2012).

MOS are generally produced via the enzymatic hydrolysis of LBG, KG, and GG due to the higher extraction efficiency, environmental benefits, lower cost, and easy processing (Yang et al. 2019). Based on present reports on the preparation of MOS, several enzymes, such as mannanases, mannosidases, galactosidases and glucosidases, must work in synergy to achieve the desired degree of polymerization (Malgas et al. 2015). Among these, endo-β-1,4-mannanase is a key enzyme for cutting off the random β-1,4-mannosidic linkages in mannan polysaccharides for MOS preparation (Srivastava et al. 2017a, b).

Based on sequence similarities and structural properties, mannanases can be classified into glycoside hydrolase (GH) families 5, 26 and 113 (Zhang et al. 2008). These enzymes have distinct modes of substrate recognition, but the molecular mechanisms governing their substrate preference and mode of action are still not fully understood. The majority of currently used mannanase-producing microorganisms such as *Bacillus* sp. (Zhang et al. 2008; David et al. 2018; Eom et al. 2016), *Klebsiella* sp. (Pongsapipatana et al. 2016) and *Aspergillus* sp. (Shimizu et al. 2015) cannot meet the increasing demand for food-grade MOS products. Although a number of studies focused on the identification of new β-mannanases to prepare MOS from one or two kinds of mannans, there are only a few reports on β-mannanases belonging to the GH113 family that can effectively hydrolyze LBG, KG, and GG for the preparation of MOS (Cao et al. 2018; Yang et al. 2019, 2015; Li et al. 2017). Furthermore, finding mannanases with high activity and stability during the catalytic process is essential for industrial application in MOS production. Therefore, mannanases which have specific characteristics, such as higher activity, acid resistance and thermal stability, are currently required (Yang et al. 2019).

In this work, we focused on a novel mannanase with higher catalytic activity and thermostability, which could potentially be used for industrial MOS production. We fully characterized its enzymological characteristics relevant for industrial use, and identified its key catalytic active site residues via homologous modeling and structural analysis of PcMan113. Rational design of enzyme for structure-based engineering was applied for obtaining the highly active mutant PcMT3 with desired properties. Subsequently, molecular docking and molecular dynamics (MD) simulations were then conducted to identify and explain the differences between highly active mutants and the wild-type enzyme. Our work provides the significantly improved variant PcMT3 for potential commercial use in the food and feed industries.

## Materials and methods

### Materials

The restriction enzymes, PrimeSTAR MAX DNA polymerase, and T4 DNA ligase were obtained from TaKaRa Biotechnology (Dalian, China). LBG, KG, GG and standard chemicals were purchased from Sigma-Aldrich (St. Louis, MO, USA) or Yuanye (Shanghai, China) and were of analytical grade unless stated otherwise. MOS standards (M2-M6) were purchased from Megazyme (Wicklow, Ireland). Synthetic oligonucleotides and primers were obtained from Genewiz (Suzhou, China).

### Vector construction and expression of endo-β-1,4-mannanase

The endo-β-1,4-mannanase gene (GenBank accession No. GIO63020.1) was cloned and ligated into the pQE-80L vector (Qiagen, Hilden, Germany) between the *Bam*HI and *Hin*dIII restriction sites, so that it contained a His6 tag (HHHHHH) at the *N*-terminus. The recombinant plasmid harboring the mannanase sequence was introduced into *E. coli* BL21(DE3), which were grown in lysogeny broth (LB) at 37 °C. When the OD_600_ reached 0.8, isopropyl β-D-1-thiogalactopyranoside (IPTG) was added to a final concentration of 0.5 mM to induce protein overexpression, which was continued at 16 °C overnight. After that, the cells were harvested by centrifugation at 4000*g* and 4 °C for 15 min, and resuspended in lysis buffer (20 mM Tris–HCl, pH 8.0, 20 mM imidazole, 0.5 M NaCl and 1 mM dithiothreitol). The cells were disrupted by sonication with 2 s pulse, 2 s output and 50% duty cycle for 30 min on ice.

### Protein purification and determination of molecular mass

The His-tag target protein was trapped onto 2 mL of Ni–NTA Superflow resin (Qiagen, Hilden, Germany) pre-equilibrated with 20 mL of lysis buffer. After washing the resin with buffer (20 mM Tris–HCl, pH 8.0, 40 mM imidazole, 0.5 M NaCl and 1 mM DTT), the target protein was eluted with 10 mL of elution buffer (25 mM Tris–HCl, pH 8.0, 500 mM imidazole, 0.5 M NaCl and 1 mM DTT) at 4 °C. The eluted fractions containing the target protein were pooled and concentrated by ultrafiltration (10 kDa MWCO, Millipore, Billerica, MA, USA) at 4 °C, and further purified by gel filtration using a Superdex 200 HR 10/300 column (GE Healthcare, Uppsala, Sweden). Fractions containing recombinant mannanase were concentrated by ultrafiltration at 4 °C. The purity and molecular mass of the purified mannanase was determined by sodium dodecyl sulfate polyacrylamide gel electrophoresis (SDS-PAGE) (Zhao et al. 2012). The purified protein was concentrated by ultrafiltration, and used for activity assays.

### Mannanase activity assay

Mannanase activity was measured using the previously described method of 3,5-dinitrosalicylic acid (DNS) colorimetric reported by Li and co-workers and with slight modification, and 0.5% (wv^−1^) LBG was chosen as the substrate (Akino et al. 1988). One unit of enzyme activity was defined as the amount of enzyme that converts 1 μmol substrate per min under the described assay conditions. The absorbance of colored solution was monitored at 540 nm by an ultraviolet–visible (UV–vis) spectrophotometer (Jinghua, Shanghai, China). Kinetic studies of wild-type (WT) mannanase and mutants were conducted in the same reaction system. The fitting curves of WT and mutants with LBG, GG and KG as substrates were recorded, and the *K*_m_ and *k*_*cat*_ parameters were calculated using nonlinear fitting to the Michaelis–Menten equation in GraphPad Prism 7.0 (GraphPad software, La Jolla, CA). The protein concentration was determined according to the Lowry method (Lowry et al, 1951), with bovine serum albumin (BSA) as the standard.

### Characterization of PcMan113

The optimal pH of purified PcMan113 was determined by measuring the enzyme’s activity in 50 mM glycine–HCl buffer (pH 2.2–3.5), 25 mM Na_2_HPO_4_–citrate buffer (pH 3.5–6.0), 50 mM phosphate buffer (PBS) (pH 6.0–8.0) and 25 mM Tris–HCl buffer (pH 7.5–9.0) at room temperature for 5 min. The effect of pH on protein stability was tested by incubating the purified PcMan113 protein at different pH values (pH 3.0–8.5) for 1.0 h at room temperature. The optimal temperature was determined by incubating PcMan113 at 15–65 °C. The thermostability was determined by measuring the residual activity after incubation of the enzyme at 20 to 85 °C for 30 min. The activity was then measured under standard reaction conditions. LBG, cassia gum, guar gum, and konjac powder were used to assess the substrate specificity of PcMan113. All activity assays were carried out in triplicate.

### Analysis of hydrolysis products

For the production of manno-oligosaccharides LBG, KG, and GG were used as the substrates, respectively, and were incubated with 5 μM of purified protein at 60 °C and pH 5.0 for 1.0 h. The hydrolysate was then boiled for 15 min to inactivate the β-mannanase and centrifuged at 20,000×*g* for 15 min at 4 °C to remove the insoluble fractions. Thereafter, the supernatants were measured by the above-mentioned DNS colorimetric method.

### Phylogenetic analysis

To reveal the evolutionary relationships of PcMan113 with other mannanases from various species, mannanase superfamily sequences were retrieved by blast search from a public database (http://www.ncbi.nlm.nih.gov/) and a phylogenetic tree was constructed using Molecular Evolutionary Genetics Analysis (MEGA) software (Hall. 2013).

### Structure modeling of PcMan113

Modeller 9.9.2. was used to construct a three-dimensional (3D) homology model of PcMan113 (Šali et al.^1993^). The crystal structure of mannanase from *Bacillus* sp. N16-5 (PDB ID: 7DV7, 1.4 Å) was chosen as the template, the homologous identity 62% between PcMan113 and the template. A sequence alignment between the template and PcMan113 was generated automatically using the align2d command, and the homology model was then generated via the automodel command. Each model was first optimized, and the structure was refined using simulated annealing in MD simulations (Abraham et al. 2015). The best model was chosen based on the values of the Modeller objective function and the DOPE assessment scores. The optimal model structure was visualized and analyzed using PyMOL software (http://www.pymol.org) (Humphrey et al. 1996).

### Site-directed mutagenesis

To generate mannanase mutants with improved characteristics, site-directed mutagenesis was conducted with the PcMan113 expression plasmid as the template using the KOD-Plus-Mutagenesis kit (Toyobo, Japan). The mutations were confirmed by DNA sequencing. PcMan113 mutants were expressed and purified as described for the WT.

### Molecular dynamics simulations and calculations

An external force was applied to the substrates center of mass along a predefined direction using steered molecular dynamics (SMD) simulations. The protein–substrate complex in Gromacs 5.1.2 software was created using the Gromos 96 53A6 force field. Subsequently, we obtained the mannobiose substrate parameters in the GROMOS96 53a6 force field from the Automated Topology Builder and Repository 2.0 webserver (https://atb.uq.edu.au/) (Koziara et al. 2014). The PcMan113–substrate complex and water molecules were placed into the simulation system, after which energy minimization was applied for the relaxation of the simulation system. After that, the constant-velocity SMD simulations were implemented, and the substrate center was pulled with a velocity of 0.01 Å ps^−1^ and a spring constant of 1000 kcal mol^−1^ Å^−2^. The values of the PcMan113-WT and mutants were acquired by calculating an asymmetric distribution of sampling windows over the distance along the substrate-binding channel.

### Statistical analysis

All experiments were performed in triplicate. One-way analysis of variance (ANOVA) was used as a test for statistical significance, with a cut-off value of *p* = 0.05.

## Results and discussion

### Sequence analysis and identification of PcMan113

A novel endo-1.4-β-mannanases named as PcMan113 was found in a proprietary database derived from a *Paenibacillus cineris*. The PcMan113 sequence was 987 bp, encoding a protein of 329 amino acid residues with a pI and molecular weights predicted to be 4.54 and 39.6 kDa, respectively. The protein showed 65% similarity with a well characterized endo-1,4-β-mannanase from *Bacillus* sp. N16-5 (Liu et al. 2021). BLAST searches against the NCBI nonredundant and CAZy databases (Lombard et al. 2014), combined with conserved domain analysis, implied that PcMan113 belongs to the GH113 family. A phylogenetic tree was constructed based on the amino acid sequences of several similar proteins and representative β-mannanases form the GH 5, GH 26, and GH 113 families using the neighbor joining method, which further revealed that PcMan113 clustered with the GH113 like β-mannanase BaMan113A (Additional file 1: Fig. S1).

**Fig. 1:**
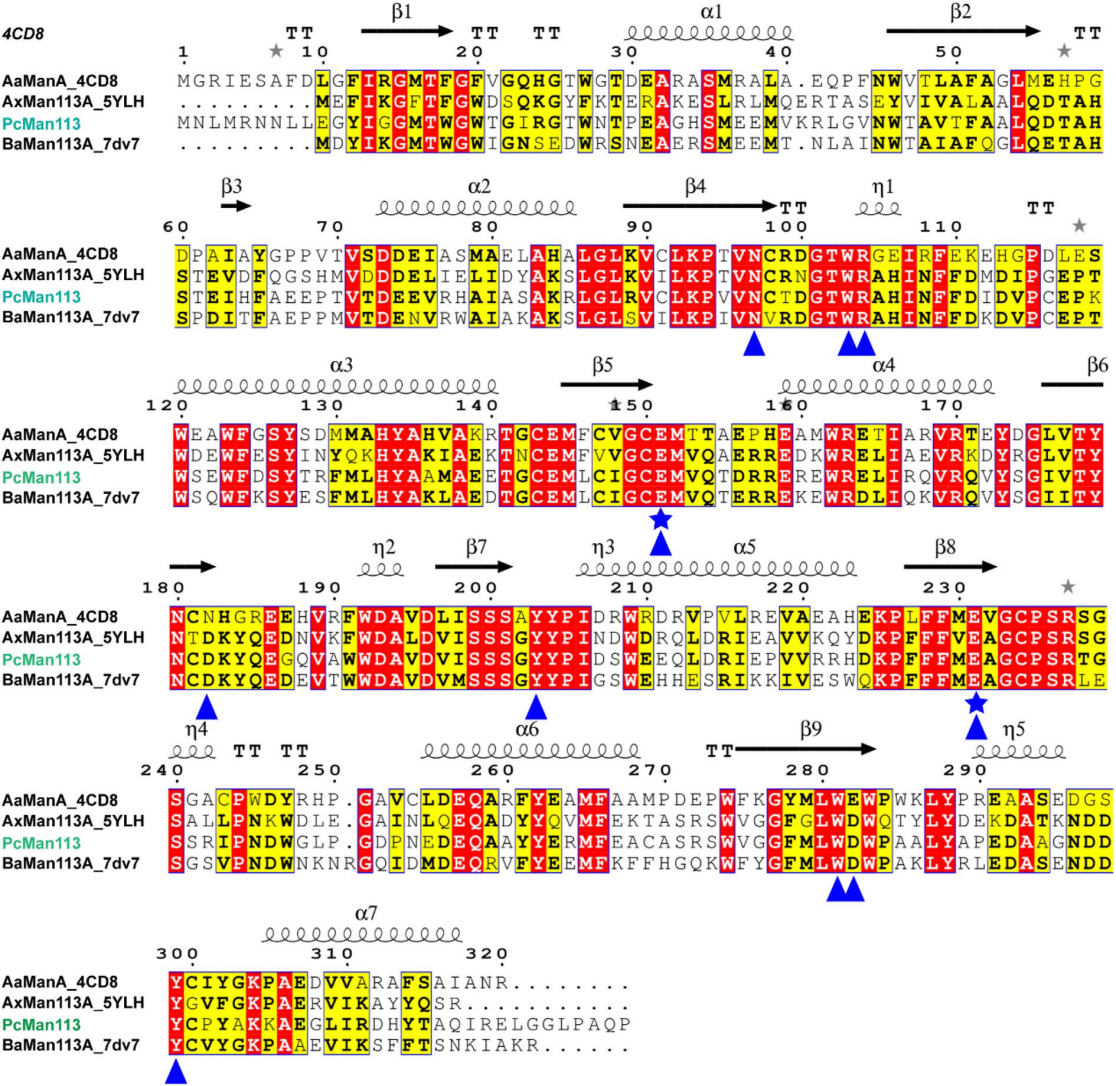
Multiple sequence alignment of PcMan113 from *Paenibacillus cineris* with other reported β-1,4-mannanase belonged to GH family 113 members (AaManA_4CDB, AxMan113A_5YLH and BaMan113A). The secondary structure of AaManA (from the PDB entry 4CDB) is shown as arrows for *β-strands* and *h* for helices. Identical and similar residues are shaded color of red and yellow, respectively. The two catalytic residues (E152 and E232) and other eight proposed substrate-binding sites (N98, W104, R105, D183, Y204, W282, D283, Y300) are marked by blue stars and triangle, respectively

PcMan113 was therefore identified as a novel member of GH family 113 with low sequence identity. The PcMan113 was predicted to be an intracellular enzyme according to SignalP analysis and it had no signal peptide. Sequence alignment with other identified GH113 family mannanases showed that several conserved residues (Fig. 1), and two catalytic residues (proton donor, E152; nucleophile, E232) and eight chemical substrate-binding site residues (N98, W104, R105, D183, Y204, W282, D283, Y300) were strictly conserved among the GH113 family members.

### Expression, purification and identification of PcMan113

**Fig. 2:**
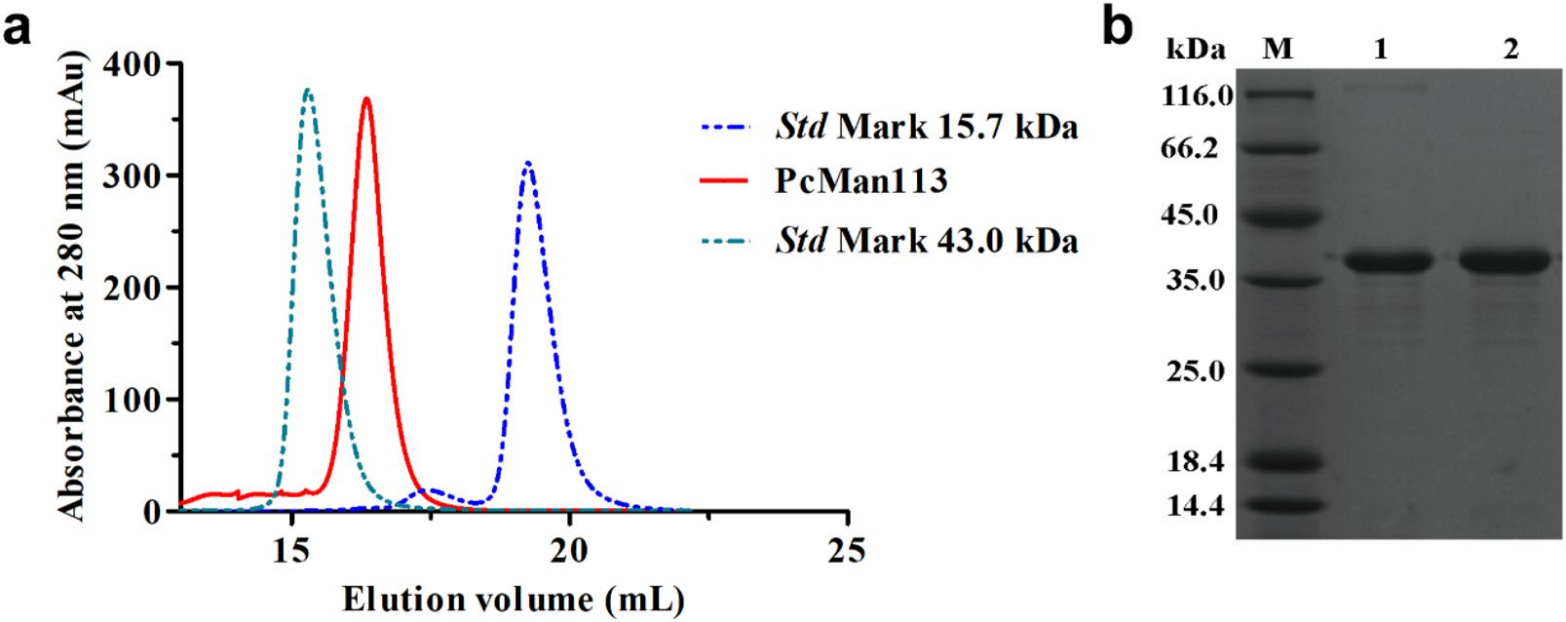
Purification of PcMan113 by size-exclusion chromatography (**a**) and SDS-PAGE analysis of purified recombinant PcMan113 (**b**). *Lane M:* marker; *lane 1–2:* the PcMan113 purified by size-exclusion chromatography

PcMan113 was successfully overexpressed in *E. coli* BL21(DE3) and purified using Ni–NTA affinity chromatography. On size-exclusion chromatography, PcMan113 eluted as a single peak between the standard protein markers rabbit muscle actin (15.7 kDa) and conalbumin (43.0 kDa) (Fig. 2a), which indicated that purified PcMan113 was a monomer in solution. Based on these results and the SDS-PAGE, which revealed a single protein band, the mass of the purified protein was in good agreement with the predicted molecular weight of 39.6 kDa (Fig. 2b).

### Characterization of purified PcMan113

**Fig. 3:**
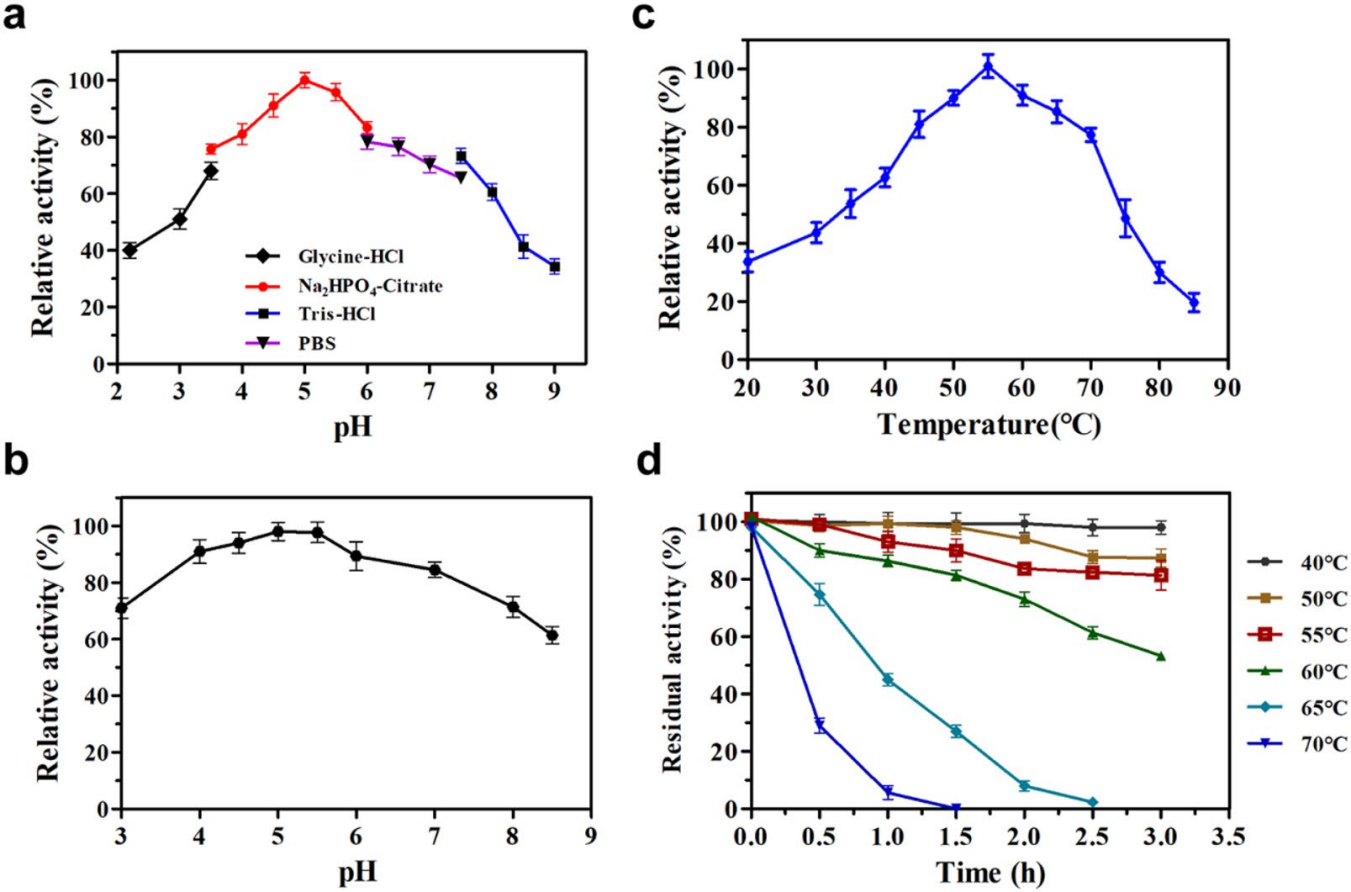
Effects of pH and temperature on the activity of PcMan113. **a** pH dependence of PcMan113. Activity was measured in the reaction mixtures adjusted to various pH values with glycine–HCl buffer, Na_2_HPO_4_–citrate buffer, PBS buffer and Tris–HCl buffer. **b** Analysis of pH stability of PcMan113. PcMan113 was preincubated at different pH values for 1 h at room temperature, and the residual activity was determined at various pH conditions. **c** Temperature dependence of PcMan113. Activity was measured at various temperatures in the standard assay conditions. **d** Thermostability analysis of PcMan113. PcMan113 was preincubated at various temperatures for 30 min. The activities of wild-type PcMan113 are represented as 100 in optimal reaction conditions, error bars are standard deviations (*n* = 3)

With LBG as the substrate, PcMan113 exhibited optimal activity in Na_2_HPO_4_–citrate buffer at pH 5.0 (Fig. 3a) and maintained more than 80% of its maximal activity at pH values between 4.0 and 7.0. Moreover, the enzyme also showed considerable stability over an extensive pH range from 3.0 to 8.0, retaining over 60% of its initial enzyme activity after incubation without substrate at room temperature for 1 h (Fig. 3b). These results indicate that PcMan113 is a moderately acidophilic enzyme, which gives it characteristic has a good application prospects in many bioconversion processes.

The temperature–activity profile indicated that PcMan113 was a typical mesophilic hydrolase, exhibiting the highest activity at 55 °C (Fig. 3c) and thermostability at 40 °C in Na_2_HPO_4_–citrate buffer. It retained more than 60% relative activity at temperatures ranging from 40 to 65 °C (Fig. 3d). However, when incubated at 70 °C, the enzyme activity declined rapidly.

The effects of metal ions and the chelator EDTA on purified PcMan113 were explored by incubating the enzyme at 55 °C for 1.0 h. The results showed that 5 mM Zn^2+^ could slightly enhance the enzyme activity to 115%, while Cu^2+^ and Ag^+^ greatly inhibited the enzyme activity. Moreover, Mn^2+^ and Fe^2+^ reduced the enzyme activity by approximately 10%, while EDTA resulted in severe inhibition (Additional file 1: Fig. S2).

### Substrate selectivity and kinetic properties of PcMan113

To determine the substrate selectivity of PcMan113, we incubated purified PcMan113 with various substrates including LBG, KG, GG, sodium carboxymethyl cellulose (SCC), beechwood xylan (BX), mannobiose (M2), mannotriose (M3), mannotetraose (M4), mannopentaose (M5) and mannohexaose (M6). The purified PcMan113 showed maximal activity towards M5 (8.2 × 10^3^ U/mg), followed by M4 (7.5 × 10^3^ U/mg), M6 (6.9 × 10^3^ U/mg), M3 (3.2 × 10^3^ U/mg) and M2 (2.6 × 10^2^ U/mg). The purified PcMan113 showed much lower activity towards KG (68.2 U/mg), LBG (31.7 U/mg) and GG (2.1 U/mg) orderly (Table1), and no activity was observed with SCC or BX. The specific activity of PcMan113 toward KG was higher than toward LBG or GG, which was consistent with other enzymes from the GH 113 family, such as BaMan113A from *Bacillus* sp. N16-5 (Liu et al. 2021), as well as AaManA (David et al. 2018) and Man113A from *Alicyclobacillus* sp. strain A4 (You et al., 2018). By contrast, mannanases from GH families of 134, 26 and 5, such as AoMan134A (Hogg et al. 2003), CrMan26 (Mandelli et al. 2020), and RmMan5A (Song et al. 2018), exhibit a high preference for LBG. PcMan113 and other GH family mannanases have different preferences toward LBG and KG, which are related to structural differences in space for accommodating the substrate with galactose side chains in a non-catalytic binding mode (Jin et al. 2016; Kumagai et al. 2015; Le et al. 2005).

**Table 1:** Substrate specificity of PcMan113

Substrate	Relative activity^a^ (100%)	Specific activity^b^ (u/mg)
LBG	100	31.7 ± 0.13
KG	2.9 × 10^2^	68.2 ± 0.36
GG	8.5	2.1 ± 0.07
SCC	ND	ND
BX	ND	ND
M2	0.9 × 10^3^	2.6 × 10^2^ ± 12.1
M3	2.1 × 10^4^	3.2 × 10^3^ ± 30.8
M4	3.5 × 10^4^	7.5 × 10^3^ ± 65.4
M5	3.9 × 10^4^	8.2 × 10^3^ ± 48.0
M6	3.3 × 10^4^	6.9 × 10^3^ ± 78.6

*ND* not detected

^a^The relative activity of PcMan113 towards LBG was set as 100%

^b^The specific activity of substrates by PcMan113 catalyzing in vitro. Reaction conditions: 5 mM substrate with 0.2 mg mL^−1^ purified protein in 200 μL PBS–citric acid buffer (20 mM, pH 6.0) at 45℃ for 10 min

The kinetic properties of purified PcMan113 were analyzed using manno-oligosaccharides as substrates, including mannobiose (M2), mannotriose (M3), mannotetraose (M4), mannopentaose (M5) and mannohexaose (M6). The kinetic parameters of PcMan113 for these substrates were calculated by nonlinear fitting to the Michaelis–Menten equation (Table 2) (Kumagai et al. 2015) The *K*_*m*_ values for M2, M3, M4, M5 and M6 were 66.1, 35.4, 5.1, 4.7 and 8.2 mM, respectively, indicating that PcMan113 has the highest affinity for M5, followed by M4 and M6. Moreover, PcMan113 showed higher catalytic efficiency with M5 (6.77 mM^−1^ s^−1^) and M4 (5.19 mM^−1^ s^−1^) than with M6, and the lowest catalytic efficiency with M2 (0.035 mM^−1^ s^−1^).

**Table 2:** Kinetic analysis of PcMan113 activity on different manno-oligosaccharides

Substrate	*K*_m_ (mM)	*k*_cat_ (s^−1^)	*k*_cat_/*K*_m_ (mM^−1^/s^−1^)
M2	66.1 ± 5.7	2.36	0.035
M3	35.4 ± 4.1	7.38	0.20
M4	5.1 ± 3.4	26.48	5.19
M5	4.7 ± 0.5	31.83	6.77
M6	8.2 ± 0.8	26.88	3.28

It was reported that GH113 family members showed considerable transglycosylation activity and could hydrolyze manno-oligosaccharides with degrees of polymerization (DP) above 3 (Zhang et al. 2008; Xia et al. 2016). Our results indicated that the smallest substrate of PcMan113 was M3 (Tables 1 and 2), which was consistent with the catalytic properties of other GH113 family members. Compared to other endo-β-mannanases, PcMan113 showed poor catalytic efficiency on M2 (0.035 mM^−1^ s^−1^) and M3 (0.20 mM^−1^ s^−1^), and the catalytic efficiency toward M3 much lower than that of Man113A (6.54 mM^−1^ s^−1^) (Xia et al. 2016) and PaMan5A (26.67 mM^−1^ s^−1^) (Couturier et al. 2013). The enzyme hydrolyzed M2 slowly (data not shown), suggesting that the hydrolysis products of PcMan113 would be mainly M2 and mannose, which makes it suitable to degrade mannans.

### Structural homology analysis and active site screening of PcMan113 protein

**Fig. 4:**
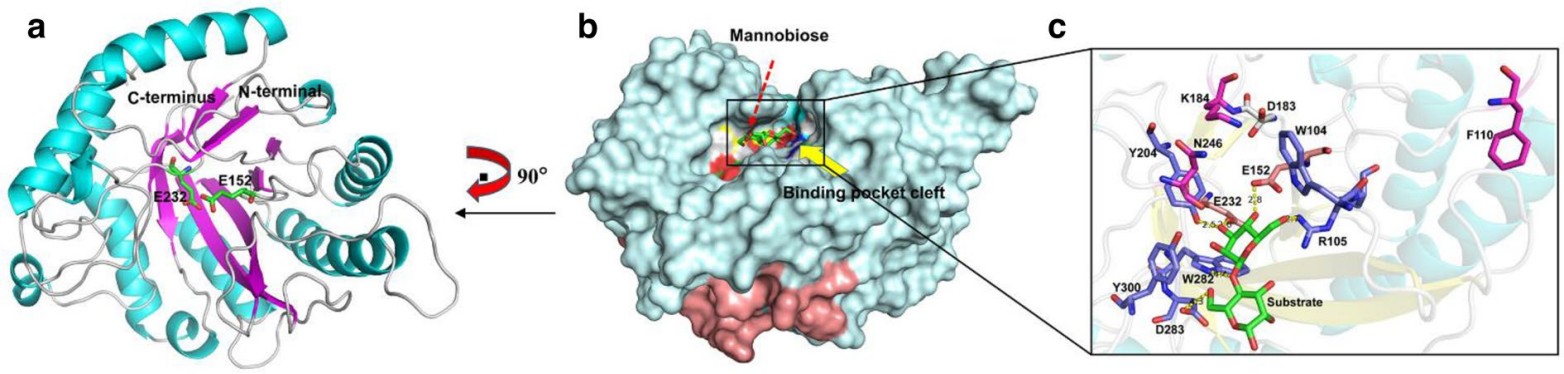
Structural homology analysis of the PcMan113–substrate complex. **a** Cartoon representation of the overall structure of PcMan113. Helices are marked by color cyan, β-sheets in magenta, and loops in white. **b** Surface representation of the overall structure of PcMan113–mannobiose complex. **c** Enlarged insight of the catalytically substrate-binding pocket sites. The two catalytic residues are marked as orange sticks. The two residues are shown in cyan sticks. The substrate mannobiose in **b** and **c** is shown as green stick, respectively. Other proposed substrate-binding sites are showed light blue sticks (W104, R105, Y204, W282, D283 and Y300), magenta sticks (F110, K184 and N246), grey stick (D183), respectively

A structural homology model of PcMan113 was generated using BaMan113A (PDB ID: 7DV7) as the template. The Ramachandran plot indicated that 97% of the residues were in the favored and allowed regions, indicating that the model of PcMan113 is plausible. PcMan113 possesses two polypeptide chains in an asymmetric unit, each folding into a typical (β/α) 8TIM-barrel architecture (Fig. 4a and Additional file 1: Fig. S3), which is consistent with the typical features of GH113 family enzymes (You et al., 2018). The catalytic residues E152 and E232 are located at the center of the β-barrel, which forms a hydrophobic area near which a deep cavity is observed. The PcMan113 may have a semi-enclosed substrate-binding pocket cleft, which was occupied by amino acids residues such as N98, W104, R105, F110, D183, Y204, W282, D283, Y300, which formed the substrate-binding site of PcMan113 (Fig. 4b and Additional file 1: Fig. S4).

Thus, this cavity accommodates the substrate and these residues interact with the substrate. The inter-molecular interface involves four regions, T58-H60, F110-E118, V154-Q155, and K184-Q186, which are located in loops (Additional file 1: Fig. S5). Among them, loop F110-E118 might act as a lid that seals the active site, which has an effect on the entrance of substrates and release of products.

### Rational engineering of PcMan113 enzyme

**Fig. 5:**
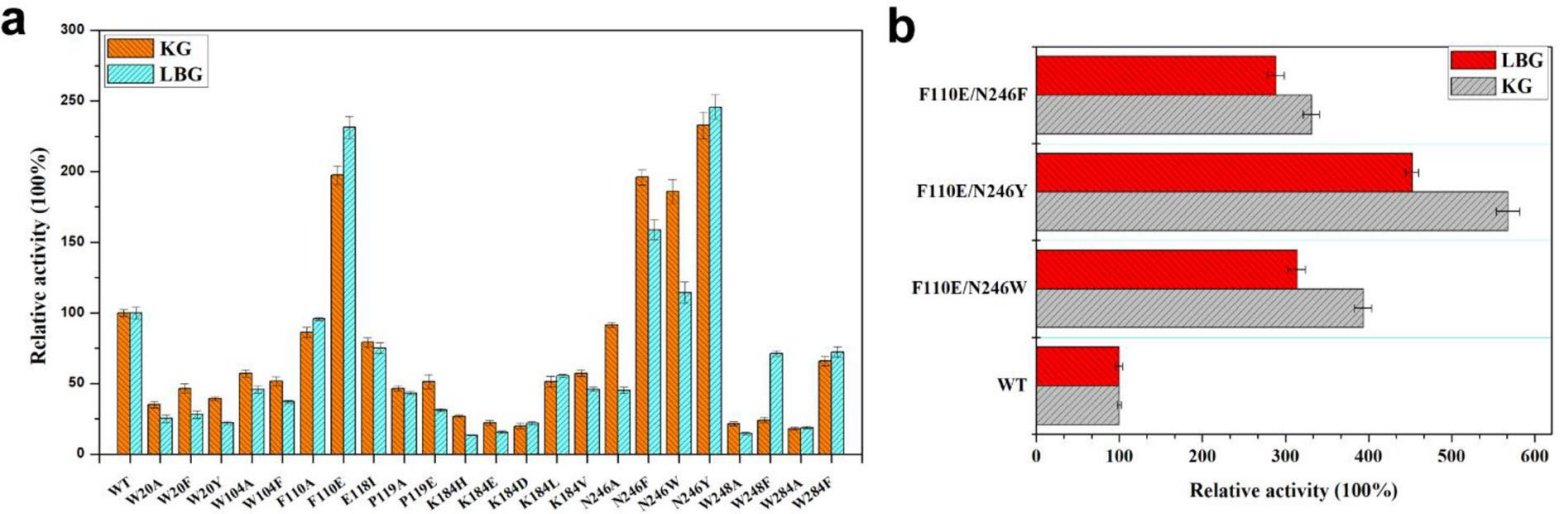
The catalytic relative activity of PcMan113-WT, mutants (**a**) and screened double mutants (**b**) towards KG and LBG. The catalytic activity of WT-PcMan113 against KG and LBG is represented as 100% and the error bars are standard deviations (n = 3)

Based on the discussed structural features of PcMan113 enzyme, a single point mutant library containing 23 single mutants at 9 residues was constructed and evaluated for activity towards the industrial substrates KG and LBG (Fig. 5a). Mutation of residues W20, W104, E118, P119, K184, W248 and W284 reduced the enzyme activity, indicating these residues played important roles in substrate binding. By contrast, mutation of residues F110 and N246 enhanced activity towards substrates of KG and LBG, and these two residues were selected as targets for site-directed mutagenesis.

On the basis of substrate-binding pocket analysis, the six mutants F110A, F110E, N246A, N246F, N246W and N246Y were constructed and evaluated. Among them, the four mutants F110E and N246F/W/Y showed higher catalytic activity towards KG and LBG than wild-type PcMan113 (WT) (Fig. 5a). The mutant F110E (PcMT1) was purified to analyze its enzyme activity (Additional file 1: Fig. S6) which showed respective 2.0- and 2.2-fold increases of catalytic activity toward KG and LBG compared with the WT. PcMT1 showed high substrate affinity and catalytic efficiency with a *K*_m_ of 1.40 mM and a *k*_cat_ of 35.20 s^−1^, respectively (Additional file 1: Table S1). The biochemical data revealed that the F110-E118 loop may affects enzymatic hydrolysis by interacting with F110 and E118 from the adjacent monomers. In addition, the loop F110–E118 was proposed to interact with the substrate and active sites, and F110 was mutated to the acidic residue glutamate or nonpolar residues with small side chains, which may affect substrate recognition and enzymatic hydrolysis.

Mutating N246 to aromatic residues led to a slight reduction in enzyme activity (Fig. 5a), and some studies reported that Trp-mediated binding plays an important role in β-mannanses of GH113 (Kumagai et al. 2015; Xia et al. 2016; Liu et al. 2021), suggesting that the stacking interaction at this position is conducive to improving enzyme activity. Therefore, N246 was replaced with Phe, Trp and Tyr in three different mutants. The most remarkable results were observed for purified N236Y (PcMT2) (Additional file 1: Fig. S6), which exhibited 233 and 246% relative activity with KG and LBG, respectively (Fig. 5a). The enzyme kinetic parameters of PcMT2 were significantly enhanced, which much higher substrate affinity and catalytic efficiency on M5 (*K*_m_, 1.8 mM; *k*_cat_/*K*_m_, 19.30) compared with the WT (Additional file 1: Table S1). These results were in agreement with previous reports that improving stacking interactions between the substrate and protein at the position of N246 could significantly enhance the catalytic activity (Liu et al. 2021; You et al. 2018; Xia et al. 2016).

Furthermore, the mutations with improved activity on KG and LBG were combined, resulting in three additional double mutants of PcMan113 (F110E/N246F, F110E/N246Y and F110E/N246W). The enzyme activity measurements reveled that all double mutants had much higher hydrolysis activity towards LBG and KG than the WT and the corresponding single point mutants (Fig. 5b). The F110E/N246Y mutant (PcMT3) was further purified for enzyme activity measurements (Additional file 1: Fig. S6). This variant retained more than 60% relative activity at temperatures ranging from 40 to 60 °C (Additional file 1: Fig. S7), exhibiting the highest thermostability at 40 °C in Na_2_HPO_4_–citrate buffer. However, when incubated at 65 °C, the enzyme activity deteriorated rapidly. The thermostability of PcMT3 was slightly reduced compared with WT-PcMan113. Moreover, compared with reported GH113 mannanases, such as Man113A (maintained activity at 40 °C) (Xia et al. 2016), AxMan113A (maintained activity below 45 °C) (You et al. 2018) and BaMan113A (maintained activity at 35 °C) (Liu et al. 2021), PcMT3 showed better thermostability at temperatures ranging from 40 to 60 °C after incubation of 2 h, which indicated that PcMT3 would has great potential for industrial applications.

In addition, PcMT3 not only exhibited a great improvement in hydrolytic activity (4.6- and 3.5-fold respective increases with KG and LBG), but also showed higher substrate affinity and catalytic efficiency (*K*_m_, 0.9 mM; *k*_cat_/*K*_m_, 37.44) with M5 than the WT enzyme (Fig. 5b and Additional file 1: Table S1). However, these results were different from other GH113 family mannanases, such as the BaMan113A mutant, which exhibited reduced substrate affinity and catalytic efficiency (Liu et al. 2021). Thus, PcMT1, PcMT2 and PcMT3 had clearly increased activity in the hydrolysis of mannans and manno-oligosaccharides, which indicated that the substrate preference of the enzyme has not been altered. At the same time, these data also demonstrated that PcMan113 hydrolyzed substrates in a standard endo-acting mode, indicating that PcMan113 can degrade mannans more completely, which was similar to the reported enzyme Man113A (Xia et al. 2016).

### MD simulations and structural analysis of the PcMT3 variant

To further explore the differences between the WT enzyme and improved variant PcMT3 in catalytic activity and protein–substrate interactions, mannobiose was docked into the binding sites of WT and PcMT3 in 30 ns MD simulations. The results indicated that the binding energy of PcMT3 with mannobiose was dramatically increased compared with the WT (− 7.85 kcal/mol vs. − 37.83 kcal/mol) (Additional file 1: Table S2). The root-mean-square deviations (RMSD) over 30 ns were calculated to investigate the stability of the WT/PcMT3–mannobiose complexes (Additional file 1: Fig. S8a). PcMan113-WT reached the equilibrium state after 15 ns of simulation, while PcMT3 was stable state after 10 ns of simulation. The average RMSD values of variants PcMT1/2/3 were 0.26 ± 0.06 nm, 0.30 ± 0.06 nm and 0.25 ± 0.04 nm, respectively, all of which were lower than that of the Pc-WT complex (0.32 ± 0.08 nm), indicating that all variants were more stable.

Moreover, the root-mean-square fluctuations (RMSF) were calculated to assess the mobility of protein residues, and the results showed that both the WT and the variants had low fluctuations ranging from 0.2 to 0.3 nm (Additional file 1: Fig. S8b). Compared with the WT, some residues in loops that are part of the substrate-binding site (such as F110 and N246) of PcMT3 had higher RMSF values, reaching > 0.4 nm in loop R243-N255, and > 0.3 nm in loop F110-E118, indicating greater flexibility in these loops than in the WT (Additional file 1: Fig. S8b). The radius of gyration (Rg) values are an indicator of the reliability of protein homology modeling, and the Rg values of WT-PcMan113 and variants all reached between1.9 and 1.95 nm (Additional file 1: Fig. S9), suggesting that the homology modeling was reliable.

**Fig. 6:**
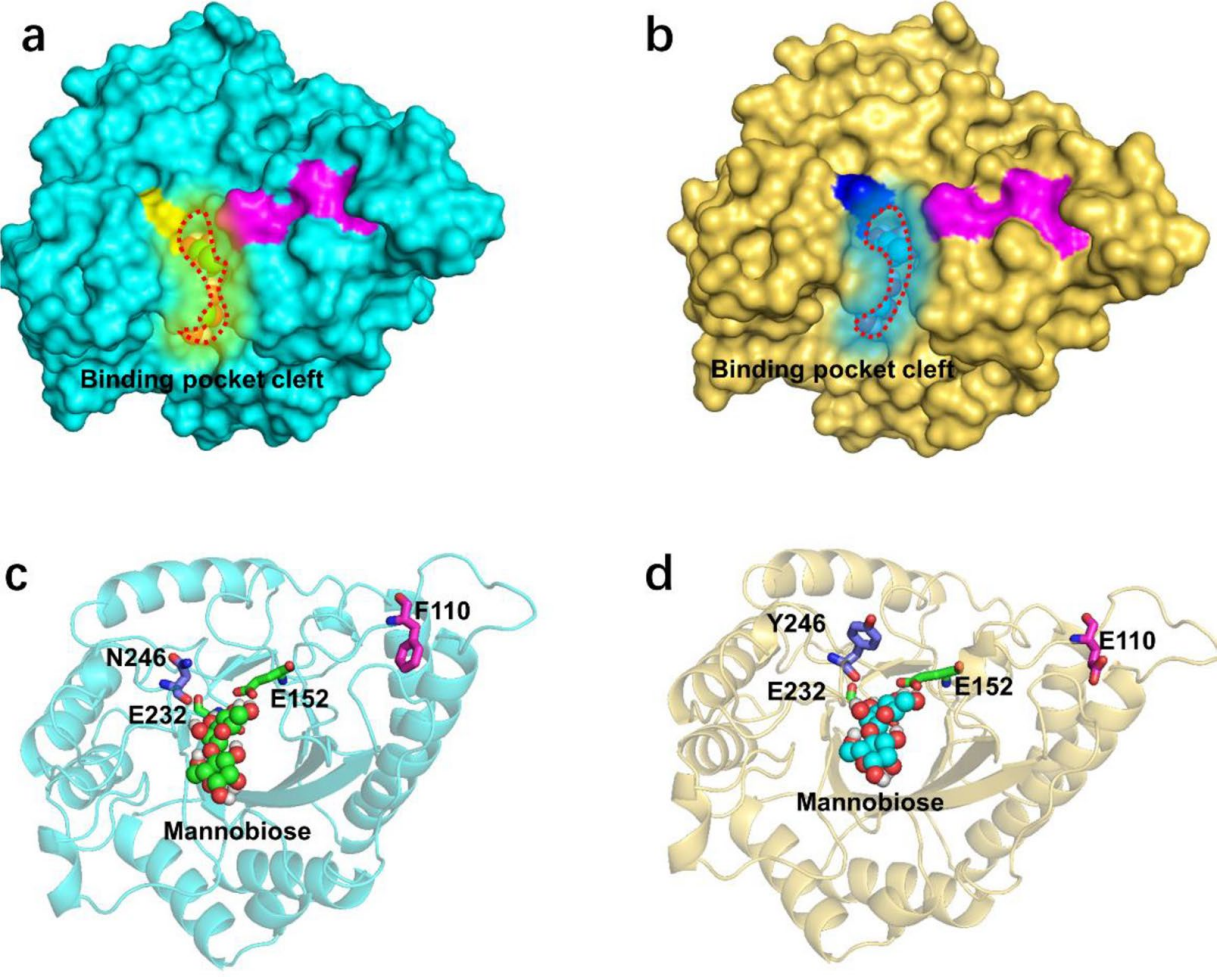
Binding modes and surface representation of the substrate in PcMan113-WT and PcMT3. The structures were obtained from cluster analysis of the MD trajectories during 30 ns of simulation. Surface representation of PcMan113-WT in color cyan (**a**) and PcMT3 in yellow (**b**) in complex with substrate mannobiose. The protein–substrate complex cartoon surface representation of PcMan113-WT in color cyan (**c**) and PcMT3 in yellow (**d**). The substrate-binding channel surface was shown with red cycles in light yellow shadow (WT) and blue shadow (PcMT3). Site N246 is shown as a yellow surface and light blue sticks ((**a**) and (**c**)), site Y246 is shown as a blue surface and sticks ((**b**) and (**d**)), site F110 and E110 are shown as a magenta surface and sticks. The substrate is presented as green spheres

**Fig. 7:**
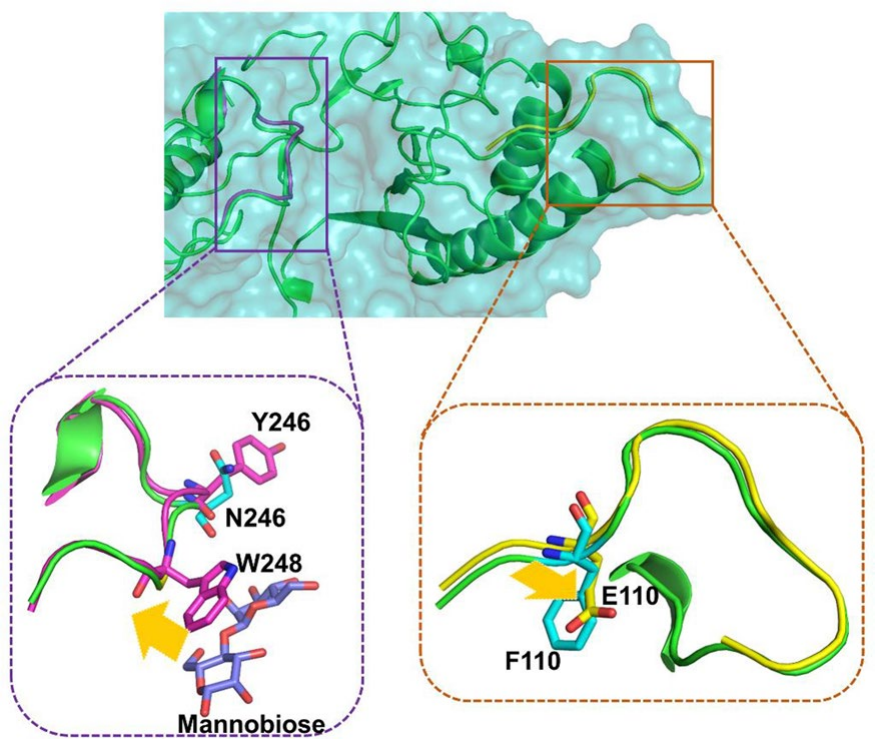
Ribbon representations of the MD-derived structures of PcMan113-WT and PcMT3 with substrate: **a** overall structural overlap of WT and PcMT3; **b** enlarged view of the loop R243-N255 (in color of magenta) in WT and MT3, and **c** enlarged view of the loop F110-E118 (in yellow) in WT and PcMT3. PcMan113-WT is shown in green and cyan, respectively. Mannobiose is shown as light blue sticks

In the WT-mannobiose complex, a narrow substrate access channel to the active site was observed (Fig. 6a). By contrast, the mutations in PcMT3 led to a somewhat bigger space in the substrate-binding channel and the substrate could easily enter the catalytic center (Figs. 6b, d and 7). Based on the previous structural analysis of the homology model and the N246Y-mediated structural effects on the substrate-binding tunnel, we hypothesized that N246 may play a pivotal role in blocking the access of the substrate into the active site, while the newly introduced Y246 side chain adds a stacking interaction for substrate binding. The N246Y mutation possibly removed steric hindrance to widen the available substrate-binding space inside the binding pocket (Fig. 6a − d). Indeed, the N246Y mutant exhibited a 46% activity improvement with LBG compared with the WT, which was also in agreement with the MD simulations (Additional file 1: Fig. S8 and Table S2). When F110 was mutated to Glu in PcMT3, its enzyme activity was further enhanced, and its RMSD indicated that the F110-E118 loop had greater flexibility (Additional file 1: Fig. S8b). We therefore hypothesized that loop F110-E118 possibly interacted with the substrate and active site (Additional file 1: Figs. S5 and S7). In addition, when site of F110 was mutated to E, E110 was found has a small side chain, which may affect substrate recognition and allow easy entrance into the binding pocket.

Furthermore, we also observed an interesting conformational change in residue W248, which was altered upon ligand binding (Fig. 7). This resulted in a more conducive conformation of the mannobiose binding channel, bringing the residue W248 that was proposed to act as a lid of the substrate-binding channel in close proximity to the substrate in the active site.

## Conclusions

The novel GH113 family endo-β-mannanase PcMan113 from *Paenibacillus cineris* was cloned, expressed, purified and characterized. Recombinant PcMan113 showed a broader substrate spectrum and better characteristics than previously reported enzymes. It exhibited the highest enzyme activity at pH 5.0 and 55 °C. It was acid tolerant, with a pH range of 3.0–8.0, and thermostable under 45 °C. Homology modeling and structural analysis of PcMan113 revealed key active site residues. Finally, we obtained the highly active mutant PcMT3 (F110E/N246Y), which not only exhibited a great improvement in hydrolytic enzyme activity (4.6- and 3.5-fold increases with KG and LBG), but also showed higher substrate affinity and catalytic efficiency (*K*_m_, 0.9 mM; *k*_cat_/*K*_m_, 37.44) with M5 than the WT enzyme. Moreover, molecular dynamics (MD) simulations revealed that the binding energy of PcMT3 with mannobiose was dramatically increased compared with that of the WT (− 7.85 kcal/mol vs. − 37.83 kcal/mol), indicating that a decrease of steric hindrance resulted in increased binding energy. A notable conformational change of residue W248 enlarged the substrate-binding channel. Our work on PcMan113 not only enriches the available GH113 family enzymes for structure-based engineering, but also provides the significantly improved variant PcMT3 for potential commercial use in the food and feed industries.

### Supplementary Information

**Additional file 1: Table S1.** Kinetic analysis of PcMan113 variants activity on different manno-oligosaccharides. **Table S2.** Binding energy of mannobiose for PcMan113 WT and PcMT3 (kcal/mol). **Fig. S1.** The phylogenetic analysis of PcMan113 proteins from different species. The amino acid sequences were subjected to Clustal W using the neighbor joining method in MEGA 4.1 (GH5-Family: mannanase belongs to glycoside hydrolase 5 family member; GH26-Family: mannanase belongs to glycoside hydrolase 26 family member; GH113-Family: mannanase belongs to glycoside hydrolase 113 family member). **Fig. S2.** The effects of mental ions and EDTA on activity of PcMan113 enzyme. PcMan113 was preincubated in Na_2_HPO_4_–citrate buffer (pH 5.0) with various metal ions (5 mM) including EDTA (5 mM) at 55 °C for 1.0 h. A reaction without the addition of any metal ions or EDTA was used as a positive control for the experiment. The activities of wild-type PcMan113 are represented as 100 in optimal reaction conditions, Error bars are standard deviations (*n* = 3). **Fig. S3**. Structural homology analysis of the PcMan113 and surface representation of the overall structure of PcMan113-mannobiose complex, E152 and E232 were shown as magenta and cyan stick, respectively. **Fig. S4.** Docking and pockets analysis of PcMan113 structure with mannobiose. Cartoon representation of the overall structure of PcMan113. Helices are marked by color cyan, β-sheets in magenta, and loops in white, the binding sites (E152, Y204, E232, D283 and Y300) were shown as light blue sticks, and substrate mannobiose was set as green stick. **Fig. S5.** Ribbon representations of the MD-derived structures of PcMan113 with substrate: overall cartoon (a) and surface (b) structural. Four small loops including T58-H60, F110-E118, V154-Q155 and K184-Q186 were shown as yellow, magenta, orange and light blue loop and surface. F110 and Mannobiose were marked with yellow a magenta stick. **Fig. S6.** SDS-PAGE analysis of purified recombinant PcMan113 mutants which were purified by gel filtration. Lane M: marker; Lane 1*:* PcMT1; Lane 2*:* PcMT2; Lane 3*:* PcMT3. **Fig. S7.** Thermostability analysis of PcMT3. PcMT3 was preincubated at various temperatures for 30 min. The activities of PcMT3 is represented as 100 in optimal reaction conditions, error bars are standard deviations (*n* = 3). **Fig. S8.** The RMSD and RMSF of mannobiose and residues over 30-ns MD simulations. Values were calculated by taking the difference from initial positions in the protein complexes. The black, red, blue and cyan lines represent the wild-type protein, PcMT1, PcMT2 and PcMT3, respectively. **Fig. S9.** The measurement of Rg for protein homology modeling WT, PcMT1, PcMT2 and PcMT3 over 30-ns MD simulations. The black, red, blue and cyan lines represent the wild-type protein, PcMT1, PcMT2 and PcMT3, respectively.

## Data Availability

The data which this article is based upon are all included in this manuscript and the additional files associated with it.

## Abbreviations

GH: Glycoside hydrolase
PcMan113: Endo-β-mannanase from *Paenibacillus cineris*
IPTG: Isopropyl-β-d-thiogalactopyranoside
WT: Wild type
LBG: Locust bean gum
KG: Konjac gum
GG: Guar gum
SCC: Sodium carboxymethyl cellulose
BX: Beechwood xylan
M2: Mannobiose
M3: Mannotriose
M4: Mannotetraose
M5: Mannopentaose
M6: Mannohexaose
MD: Molecular dynamics
RMSF: Root-mean-square fluctuations
PME: Particle mesh Ewald
RMSD: The root-mean-square deviations
Rg: The radius of gyration values
