# Supplementary material for: Reshaping the binding channel of a novel GH113 family β-mannanase from Paenibacillus cineris (PcMan113) for enhanced activity
Source: Bioresour Bioprocess. 2022 Mar 5;9(1):17. doi: 10.1186/s40643-022-00505-7 (PMC10992819; doi:10.1186/s40643-022-00505-7)
Supplement: Supplementary file 1 — Additional file 1: Table S1. Kinetic analysis of PcMan113 variants activity on different manno-oligosaccharides. Table S2. Binding energy of mannobiose for PcMan113 WT and PcMT3 (kcal/mol). Fig. S1. The phylogenetic analysis of PcMan113 proteins from different species. The amino acid sequences were subjected to Clustal W using the neighbor joining method in MEGA 4.1 (GH5-Family: mannanase belongs to glycoside hydrolase 5 family member; GH26-Family: mannanase belongs to glycoside hydrolase 26 family member; GH113-Family: mannanase belongs to glycoside hydrolase 113 family member). Fig. S2. The effects of mental ions and EDTA on activity of PcMan113 enzyme. PcMan113 was preincubated in Na2HPO4–citrate buffer (pH 5.0) with various metal ions (5 mM) including EDTA (5 mM) at 55 °C for 1.0 h. A reaction without the addition of any metal ions or EDTA was used as a positive control for the experiment. The activities of wild-type PcMan113 are represented as 100 in optimal reaction conditions, Error bars are standard deviations (n = 3). Fig. S3. Structural homology analysis of the PcMan113 and surface representation of the overall structure of PcMan113-mannobiose complex, E152 and E232 were shown as magenta and cyan stick, respectively. Fig. S4. Docking and pockets analysis of PcMan113 structure with mannobiose. Cartoon representation of the overall structure of PcMan113. Helices are marked by color cyan, β-sheets in magenta, and loops in white, the binding sites (E152, Y204, E232, D283 and Y300) were shown as light blue sticks, and substrate mannobiose was set as green stick. Fig. S5. Ribbon representations of the MD-derived structures of PcMan113 with substrate: overall cartoon (a) and surface (b) structural. Four small loops including T58-H60, F110-E118, V154-Q155 and K184-Q186 were shown as yellow, magenta, orange and light blue loop and surface. F110 and Mannobiose were marked with yellow a magenta stick. Fig. S6. SDS-PAGE analysis of purified recombinant PcMan113 mutant [file 40643_2022_505_MOESM1_ESM.docx]

Supplementary Information

**Reshaping the binding channel of a novel GH113 family β-Mannanase from *Paenibacillus cineris* (PcMan113) for enhanced activity**

Dengyue Sun^1,3^, Chao Li^2^, Pengpeng Cui^3^, Jie Zhang^3^, Yaolin Zhou^3^, Mian Wu^2^, Xia Li^3^, Teng-fei Wang^1,3^, Zhixiong Zeng ^3*^, Hui-Min Qin^1,2*^

^1^ State Key Laboratory of Biobased Material and Green Papermaking; College of Bioengineering, Qilu University of Technology; Shandong Academy of Sciences; Jinan 250100, P. R. China

^2^ College of Biotechnology, Tianjin University of Science and Technology; Tianjin 300457, P. R. China

^3^ School of Bioengineering, Qilu University of Technology, Jinan 250353, Shandong Province, PR China

E-mail: zengzx@qlu.edu.cn; huiminqin@tust.edu.cn

**Table S1**

Kinetic analysis of PcMan113 variants activity on different manno-oligosaccharides

| Enzyme | Substrate | *K*_m_  (mM) | *k*_cat_  (s^-1^) | *k*_cat_/*K*_m_  (mM^-1^/s^-1^) |
| --- | --- | --- | --- | --- |
| PcMT1 | M4 | 4.7±1.8 | 29.51 | 6.28 |
|  | M5 | 1.4±0.2 | 35.20 | 25.14 |
|  | M6 | 3.2±0.5 | 27.46 | 8.58 |
| PcMT2 | M4 | 4.5±1.7 | 26.48 | 5.89 |
|  | M5 | 1.8±0.1 | 34.75 | 19.30 |
|  | M6 | 4.3±0.4 | 25.62 | 5.96 |
| PcMT3 | M4 | 3.1±2.5 | 26.48 | 8.54 |
|  | M5 | 0.9±0.03 | 33.70 | 37.44 |
|  | M6 | 2.0±0.8 | 29.87 | 14.90 |

**Table S2**

Binding energy of Mannobiose for PcMan113 WT and PcMT3 (kcal/mol).

|  | WT | PcMT3 |
| --- | --- | --- |
| ΔG_VDW_^a^ | -58.94 | -55.09 |
| ΔG_Et_^b^ | -21.57 | -39.31 |
| ΔG_polar_^c^ | 81.42 | 71.80 |
| ΔG_Apolar_^d^ | -8.76 | -15.23 |
| ΔG_binding_^e^ | -7.85 | -37.83 |
| a: van der Waals energy; b: Electrostatic energy c: polar-solvation energy; d: non-polar solvation energy; e: ΔG_binding_=ΔG_VDW_+ΔG_Et_+ΔG_Polar_+ΔG_Apolar_ | | |


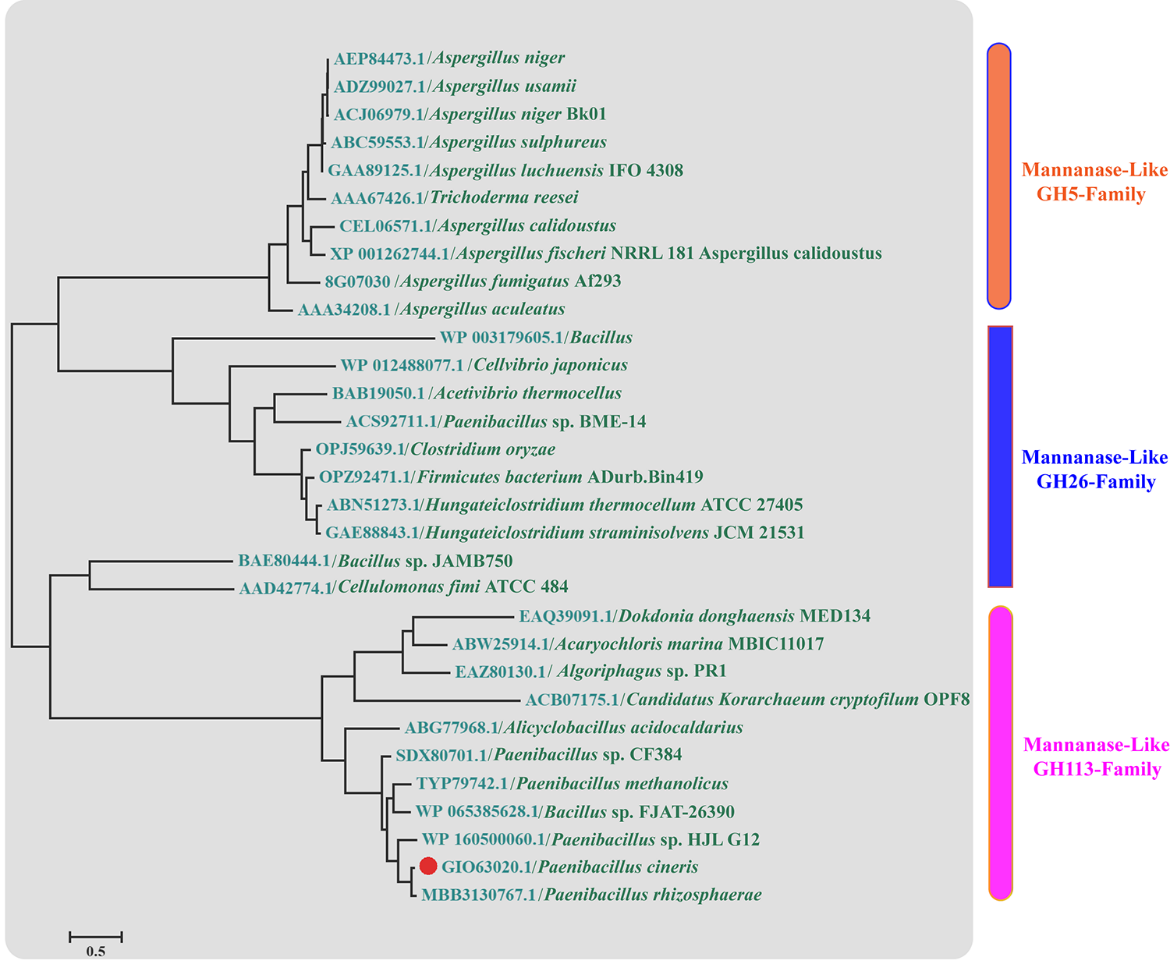


**Fig. S1** The phylogenetic analysis of PcMan113 proteins from different species. The amino acid sequences were subjected to Clustal W using the neighbor joining method in MEGA 4.1 (GH5-Family: mannanase belongs to glycoside hydrolase 5 family member; GH26-Family: mannanase belongs to glycoside hydrolase 26 family member; GH113-Family: mannanase belongs to glycoside hydrolase 113 family member).


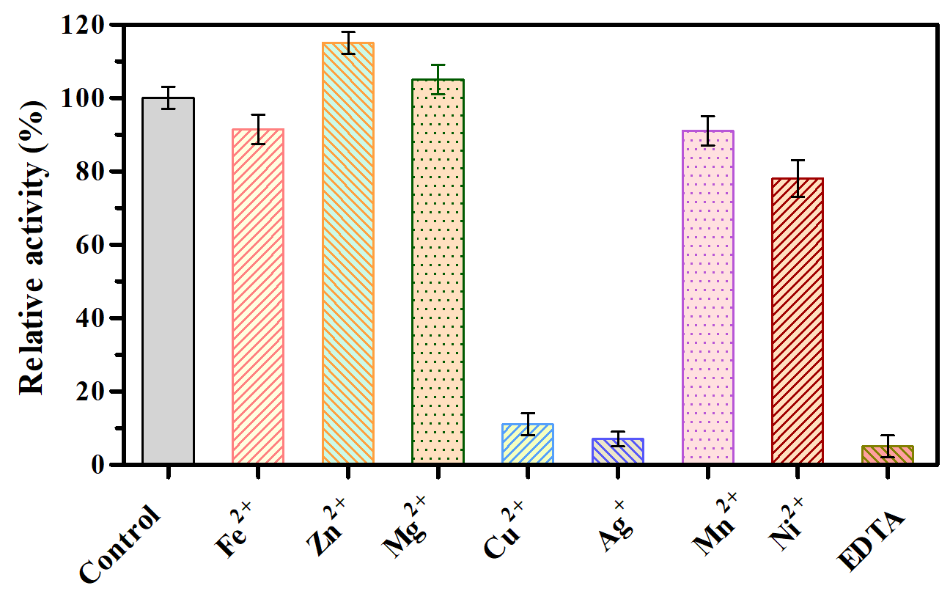


**Fig. S2** The effects of mental ions and EDTA on activity of PcMan113 enzyme. PcMan113 was preincubated in Na_2_HPO_4_-citrate buffer (pH 5.0) with various metal ions (5 mM) including EDTA (5 mM) at 55 °C for 1.0 h. A reaction without the addition of any metal ions or EDTA was used as a positive control for the experiment. The activities of wild-type PcMan113 are represented as 100 in optimal reaction conditions, Error bars are standard deviations (*n*=3).


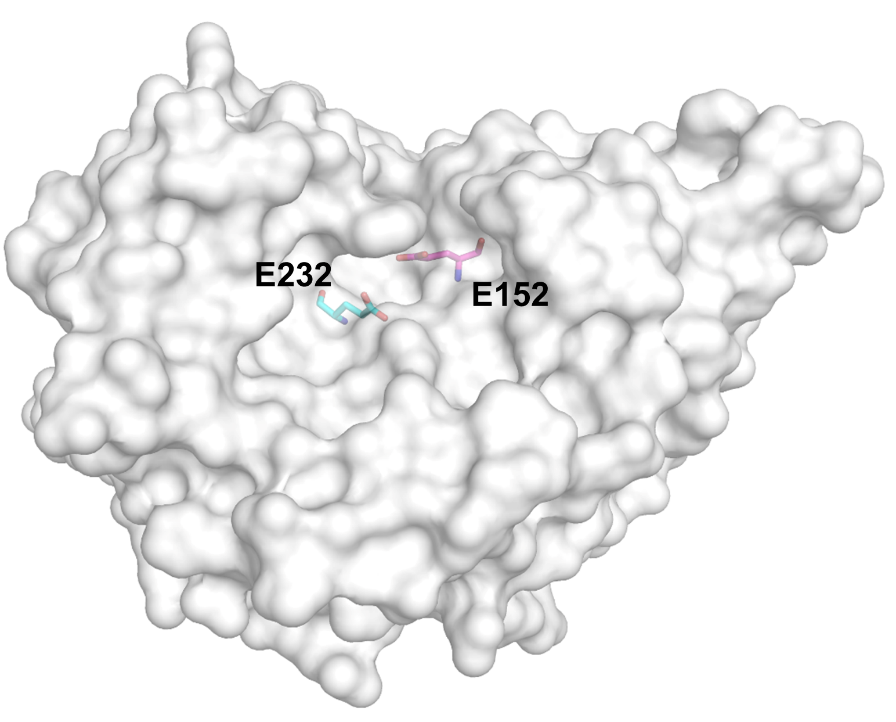


**Fig. S3** Structural homology analysis of the PcMan113 and surface representation of the overall structure of PcMan113-mannobiose complex, E152 and E232 were shown as magenta and cyan stick, respectively.


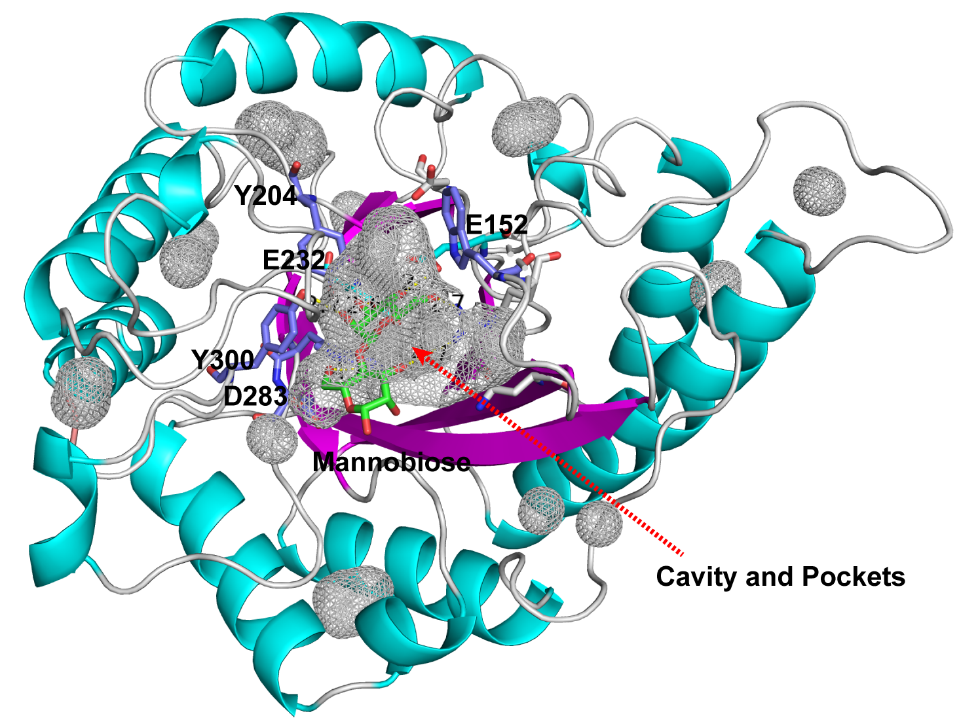


**Fig. S4** Docking and pockets analysis of PcMan113 structure with mannobiose. Cartoon representation of the overall structure of PcMan113. Helices are marked by color cyan, β-sheets in magenta, and loops in white, the binding sites (E152, Y204, E232, D283 and Y300) were shown as light blue sticks, and substrate mannobiose was set as green stick.


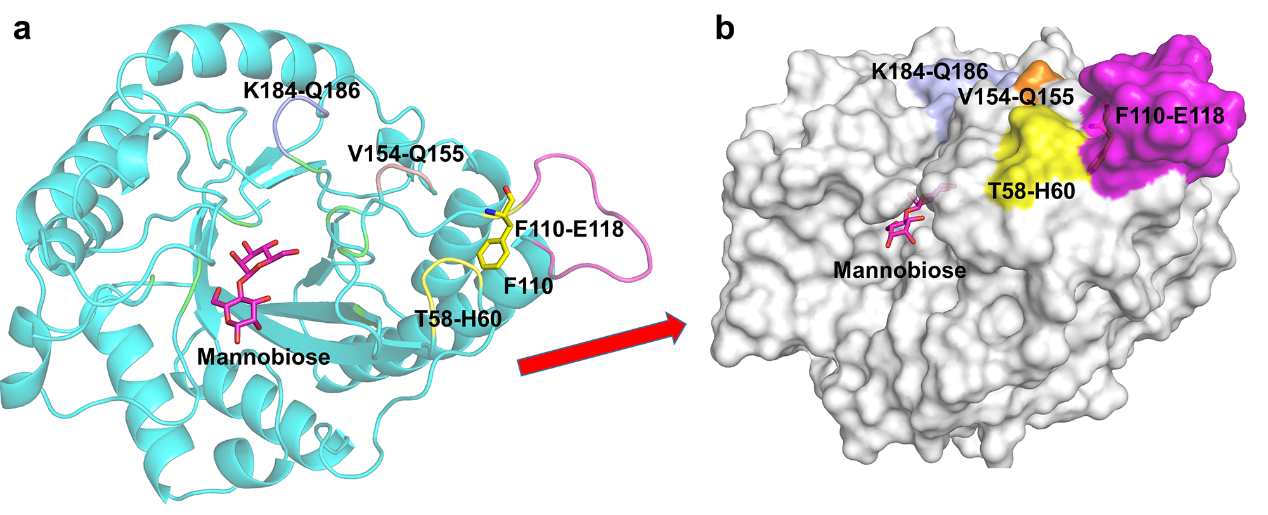


**Fig. S5** Ribbon representations of the MD-derived structures of PcMan113 with substrate: overall cartoon(a) and surface (b) structural. Four small loops including T58-H60, F110-E118, V154-Q155 and K184-Q186 were shown as yellow, magenta, orange and light blue loop and surface. F110 and Mannobiose were marked with yellow a magenta stick.


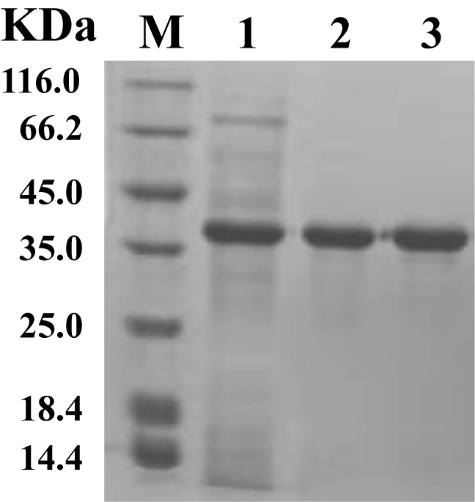


**Fig. S6** SDS-PAGE analysis of recombinant PcMan113 mutants which were purified by gel filtration. Lane M: marker; Lane 1*:* PcMT1; Lane 2*:* PcMT2; Lane 3*:* PcMT3.


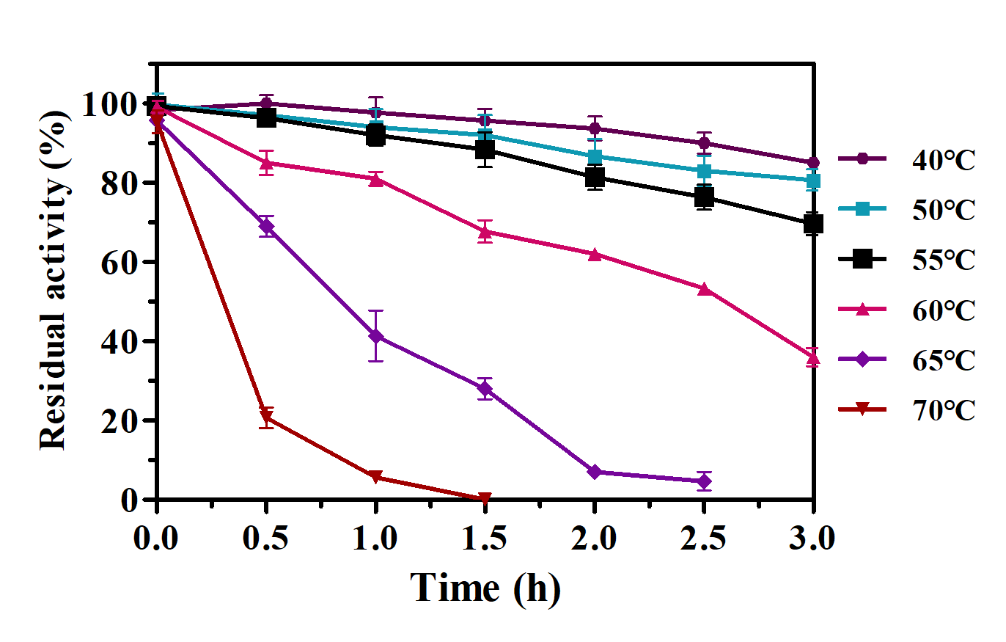


**Fig. S7** Thermostability analysis of PcMT3. PcMT3 was preincubated at various temperatures for 30 min. The activities of PcMT3 is represented as 100 in optimal reaction conditions, error bars are standard deviations (*n*=3).


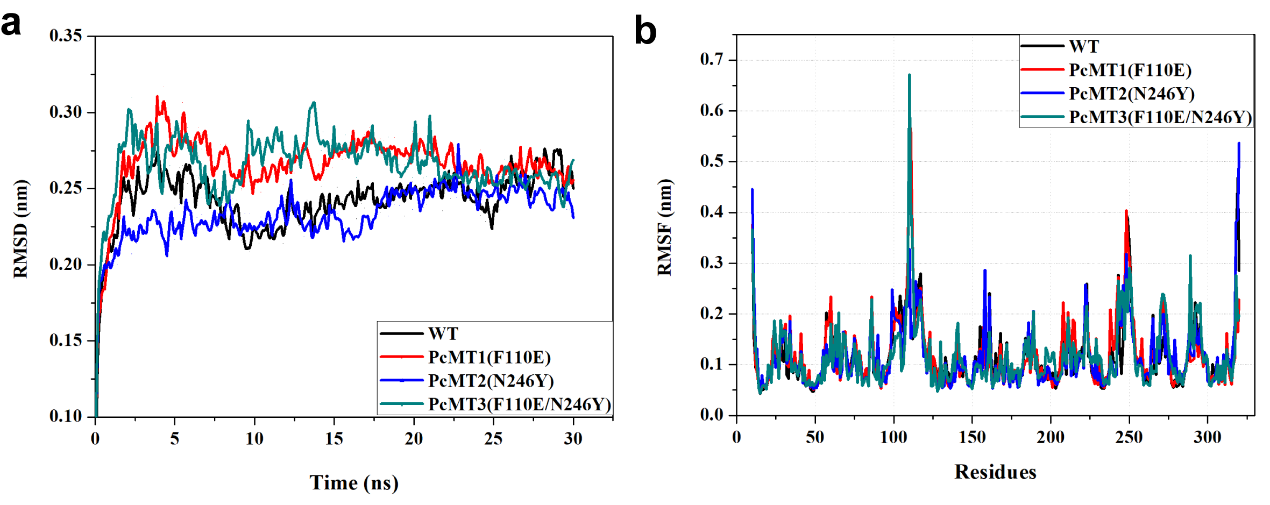


**Fig. S8** The RMSD and RMSF of mannobiose and residues over 30-ns MD simulations. Values were calculated by taking the difference from initial positions in the protein complexes. The black, red, blue and cyan lines represent the wild-type protein, PcMT1, PcMT2 and PcMT3, respectively.

**
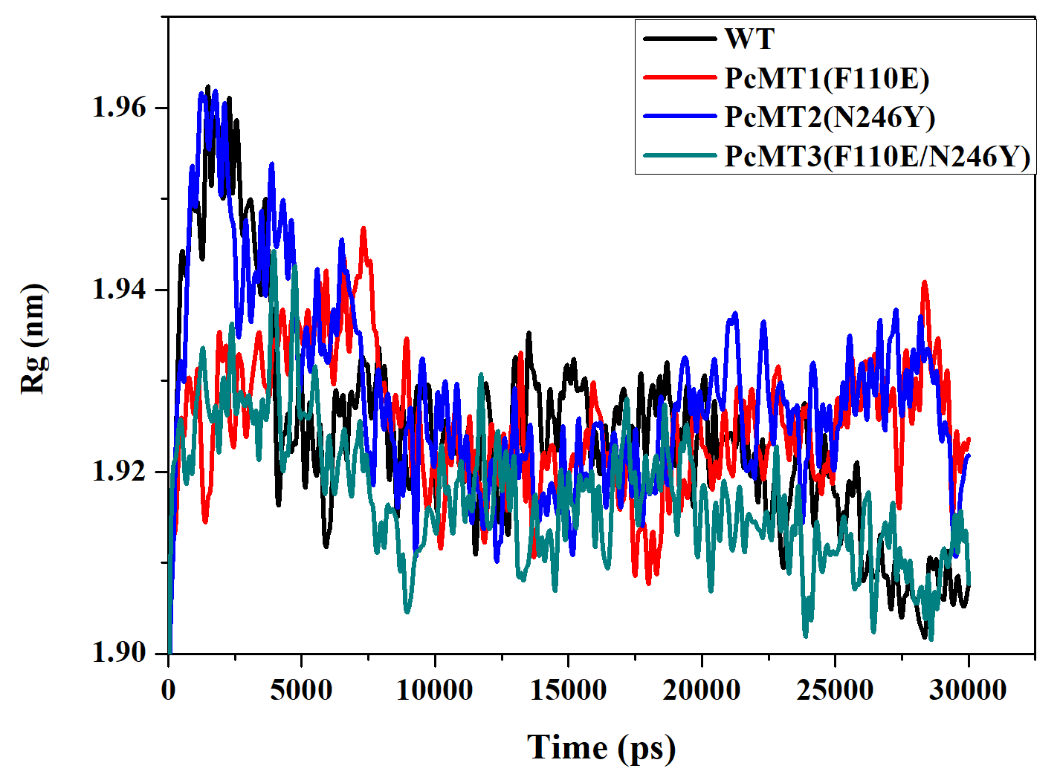
**

**Fig. S9** The measurement of Rg for protein homology modeling WT, PcMT1, PcMT2 and PcMT3 over 30-ns MD simulations. The black, red, blue and cyan lines represent the wild-type protein, PcMT1, PcMT2 and PcMT3, respectively.
